# The association of severe COVID anxiety with poor social functioning, quality of life, and protective behaviours among adults in United Kingdom: a cross-sectional study

**DOI:** 10.1186/s12888-023-04595-1

**Published:** 2023-02-21

**Authors:** Jacob D. King, Aisling McQuaid, Verity C. Leeson, Oluwaseun Samuel, Josiah Grant, Muhamad Saad Imran Azeem, Kirsten Barnicot, Mike J. Crawford

**Affiliations:** 1 Division of Psychiatry, Department of Brain Sciences, Imperial College London, Commonwealth Building, Hammersmith Hospital, W12 0NN London, UK; 2 Imperial College School of Medicine, London, UK; 3 Division of Health Services Research and Management, City University of London, London, UK

**Keywords:** Anxiety disorders, Hypochondriasis, Health behavior, COVID-19, Pandemic

## Abstract

**Background:**

Anxiety about COVID-19 is common. For most people this is an appropriate response to the loss of livelihoods and loved-ones, disruptions to social networks, and uncertainty about the future. However, for others these anxieties relate to contracting the virus itself, a phenomenon termed COVID anxiety. Little is known about the characteristics of people with severe COVID anxiety or the impact it has on their daily lives.

**Methods:**

We conducted a two-phase cross-sectional survey of people aged 18 or over who were living in United Kingdom, self-identified as anxious about COVID-19, and had a score of ≥9 on the Coronavirus Anxiety Scale. We recruited participants nationally through online adverts and locally via primary care services in London. Data on demographic and clinical factors were used in multiple regression modelling to examine the greatest contributors to functional impairment, poor health-related quality of life and protective behaviours in this sample of individuals with severe COVID anxiety.

**Results:**

We recruited 306 people with severe COVID anxiety between January and September 2021. Most were female (*n* = 246, 81.2%); they had a median age of 41 (range = 18–83). The majority of participants also had generalised anxiety (*n* = 270, 91.5%), depression (*n* = 247, 85.5%), and a quarter (*n* = 79, 26.3%) reported a physical health condition which put them at increased risk of hospitalisation with COVID-19. Half had severe social dysfunction (*n* = 151, 52.4%). One in ten reported never leaving their home, one in three washed all items brought into their house, one in five washed their hands constantly, and one in five of those with children reported not sending them to school because of fears of COVID-19. Increasing co-morbid depressive symptoms best explained functional impairment and poor quality of life after controlling for other factors.

**Conclusions:**

This study highlights the high degree of co-occuring mental health problems, and the extent of functional impairment and poor health-related quality of life among people with severe COVID anxiety. Further research is needed to establish the course of severe COVID anxiety as the pandemic progresses, and steps that can be taken to support people who experience this distress.

**Supplementary Information:**

The online version contains supplementary material available at 10.1186/s12888-023-04595-1.

## Background

The SARS-CoV2 pandemic, as in previous viral pandemics, has led to increasing numbers of people experiencing poor mental health: [1, 2] 49.6% of the adult population of United Kingdom reported elevated levels of anxiety during the British first wave, notably higher than pre-pandemic levels [3–5]. The incidence of depressive symptoms, health anxiety, substance misuse and suicidal ideation have also increased during this time [6–9]. Understandably many factors are likely to explain these patterns including the unexpected disruption to usual routines, social support structures and occupation, the bereavement and harm caused to individuals and communities, and direct physiological effects [10–12].

Fears of catching the virus and anticipation of harm coming to oneself or loved ones are also commonly reported among the world population [13]. Public health recommendations to ‘lockdown’ areas of high transmission and to follow recommended behaviours, including handwashing, avoiding ‘unnecessary’ social contacts, and regular testing are likely to substantially reduce the risk of transmission; available evidence suggests that anxiety, fear, and perceived risk from viral pandemics increase adherence with these measures [14–19]. However for some the fear of themselves or a loved one becoming infected by SARS-CoV2 is no longer adaptive, and is so great that it impairs their daily functioning [20, 21]. For some individuals with ‘COVID anxiety’, worries of contagion are overwhelming, with poor sleep, and somatic manifestations of anxiety sometimes misattributed to the disease itself [22], and may lead people to drastically exceed the recommendations for measures to reduce viral transmission, with minimal protective returns and at the expense of a satisfying life [23, 24].

In a nationally representative survey of a sample of the German population the large majority (> 70%) did not report clinically meaningful COVID anxiety, while around 5% scored highly on measures representing a severe and dysfunctional experience of COVID anxiety [25]. Research to date has found that women, both younger and older adults, those with higher personality trait neuroticism, who are living alone or with vulnerable people, and with pre-existing mental health problems are more likely to live with COVID anxiety during the pandemic [26–30]. Increasing fearfulness of COVID-19 is associated with increased rates of mental health disorders [31], and attempts to conceptualise these anxious responses have identified the contribution of generalised, social, and health anxiety, as well as obsessive-compulsive symptomatology, and several psychometric tools have been developed to capture these constructs within COVID anxiety [21, 32].

Severe anxiety disorders have well-documented impacts on daily life [33], however to date little work has reported on the lives of individuals with the most severe experiences of COVID anxiety who may benefit from the support of mental health services. Accordingly we hypothesised that a large proportion of people living with severe COVID anxiety would live with significant impacts to their social and occupational functioning, a poor quality of life, live with other mental health disorders, and would employ a number of COVID-specific protective behaviours to excess. The aims of the present study were therefore twofold. First, to characterise a sample of people with severe COVID anxiety and their demographic details, comorbid health conditions, psychopathology, and the degree of protective behaviours which are employed, and secondly, to examine the associated impact on their social and occupational functioning and quality of life. A better understanding of the mental health impacts of the COVID-19 pandemic will help to improve the support available for those suffering, and inform clinicians and policy makers on clinical and public health measures [34].

## Methods

This two-step analytic cross-sectional study uses data collected at baseline from Imperial College London’s COVID-19 Anxiety Project (CAP), a longitudinal study of people with severe COVID anxiety, a full description of which has been published elsewhere [35]. This article was written in line with STROBE guidelines for cross-sectional studies [36].

### Recruitment

An initial screening survey which targeted individuals self-identifying as having significant worries about the SARS-CoV2 pandemic was advertised nationally on social media platforms, the MQ mental health research [37] and Anxiety UK [38] websites and with *en masse* text messaging from 19 participating General Practices in Greater London. This initial survey included the Coronavirus Anxiety Scale (CAS) [21] and incorporated the project’s cohort inclusion criteria; requiring the respondent to confirm that they were aged 18 or over, a UK resident, able to self-complete the questionnaire in English, and were without a history of psychotic illness. Those meeting inclusion criteria, and scoring 9 or more on the CAS indicating severe COVID anxiety [21] were then invited by e-mail to complete the CAP baseline survey, as described below, for which they were offered an e-voucher worth 10GBP. All responses were collected between the third UK national lockdown in January 2021 until September 2021 when social distancing measures had been eased but the incidence of COVID remained high. All surveys were hosted on the Qualtrics online platform, and there was alternative provision for participants to complete the survey via telephone with one of the research team, although this was rarely used.

### Ethical approval

The COVID Anxiety Project received approval from the Leicester Central Research Ethics Committee and Health Regulation Authority in 2020, reference number 20/EM/023. Research was conducted in-line with the Declaration of Helsinki 1964, and responses were managed following General Data Protection regulations. All individuals were required to sign to consent to participate, and received a copy of their consent form.

### Measures

We assessed anxiety related to contracting COVID-19 in the initial screening survey using the 5-item Coronavirus Anxiety Scale [20, 21]. The CAS was the first major scale to measure COVID anxiety, and the only one available with validity data at the time of this study’s conception although many others have since been published. It includes physiological dimensions of anxiety, including sleep disturbance, nausea, dizziness, and gastrointestinal upset, as these were demonstrated to have the strongest construct validity from a longer list of symptoms among adults anxious about COVID. A CAS score of 9 or more was able to identify those with dysfunctional and therein severe COVID anxiety with 90% sensitivity and 85% specificity [22].

### Outcomes

We assessed social and occupational functioning using the Work and Social Adjustment Scale (WSAS), a 5-item self-administered questionnaire which has been widely used and validated in the general population and clinical samples [33, 39]. Scores greater than 10 on the WSAS indicate moderate psychopathology with associated functional impairment, and scores of 21 or greater are categorised as ‘severe functional impairment’ [39, 40].

Quality of life was assessed using the ‘EuroQuol 5-Domains’ (EQ-5D-3 L) instrument, developed for use among a wide range of health populations, where a single item for each domain - mobility, self-care, usual activities, pain/discomfort and anxiety/depression – is assessed on three ordinal ‘levels’ of increasing severity of impact [41]. For example, 1 represents ‘no mobility difficulties’ and 3 ‘severe difficulties with mobility’. We calculated EQ-5D-3 L index scores to create a continuous variable using the time trade-off valuation technique from UK population standardised scores [42]. An index score of 1 is interpreted as full health, and 0 as a health state equated to death [41, 43].

Health behaviours enacted in response to pandemics have previously been conceptualised into three translatable groups [16]: management behaviours (testing for COVID-19, consuming news media reports and social media on the subject), avoidant behaviours (not leaving the home, avoiding public transport, shopping exclusively online, not sending children to school), and preventive behaviours (washing one’s hands or groceries, mask wearing). In this study, COVID related protective behaviours were investigated using novel items co-developed early in the project in collaboration with mental health service-users who identified behaviour changes associated with fears of COVID infection as listed in Additional file 1: Appendix 1. Participants were asked to consider in the past week compared to pre-pandemic how often they 1) washed their hands 2) groceries or packages coming in to their home and 3) their clothes; 4) how often they left their home, 5) how they got food in to their home, 6) if they had children whether they were sent to school when able; and 7) how often they consumed news media about the pandemic. Five of these items were established on Likert scales, with the most severe option representing excessive behaviours or those not recommended by UK public health guidance (Additional file 1: Appendix 1). These behaviours were grouped into the pre-established pandemic protective behaviour categories with watching COVID related broadcasts representing management behaviours, not leaving the home as an avoidant behaviour, and three washing items representing preventative behaviours [16].

### Covariates

The survey collected demographic details: age, sex, UK census ethnic group, comorbid medical conditions, employment status and household composition (for analysis these were grouped into binary ‘employed’ or ‘unemployed’, and ‘alone’ or ‘with others’ categories), whether they had had COVID-19 themselves and if so whether they were hospitalised, if they had a close friend or family member hospitalised by COVID-19, who they lived with, and whether their household included someone thought to be vulnerable to COVID-19.

For the purpose of analysis, we aimed to create proxy indicators which people would be aware increased their risk from COVID-19, including male gender, increasing age, certain ethnic groups, and having an at-risk health condition [44]. Ethnicity data was grouped into binary groups of lower and higher risks of hospitalisation and mortality associated with COVID-19, based on British Office of National Statistic reports compiling data from January 2020 – December 2021 [45]. In this way people from South Asian (Indian, Bangladeshi, Pakistani), Black (Black African/Caribbean) and ‘Other’ groups were classified as being at higher risk compared to those with White British and Irish, White Other, and Chinese backgrounds.

We selected to measure a number of co-occuring mental health covariates including depression, generalised anxiety, health anxiety, obsessive-compulsive psychopathology, personality difficulty, and drug and alcohol use based on experience from a previous study of health anxiety [46]. Clinical diagnoses were not possible to facilitate, and in lieu self-report psychopathological assessment tools were used with validated cut-off scores as proxies of diagnosis. Respectively, these were the Patient Health Questionnaire 9-item (PHQ-9), Generalised Anxiety Disorder 7-item scale (GAD-7), Obsessive-Compulsive Inventory Revised (OCI-R), Health Anxiety Inventory short form (sHAI), Standardised Assessment of Personality Abbreviated Scale (SAPAS), the Alcohol Use Disorders Identification Test for Consumption (AUDIT-C) and the Single Drug-use Item (SDUI) which are outlined in detail in Table 1.

**Table 1:** Summary of psychopathology assessment tools

Scale	Outcome Measured	Possible range	Cut-off	Cut-off Sensitivity, Specificity (%)	Nature of Items	No. of items	Scale	Timescale
Coronavirus Anxiety Scale (CAS)	COVID-associated Anxiety	0–20	≥ 9 [21] Severe COVID-19 Anxiety	90, 85 [21]	Frequency of adverse somatic-anxious responses to COVID-related cognitions	5	5-point Likert- scale; Higher scores, more frequent symptom	Past 2 weeks
Short Health Anxiety Inventory (sHAI) *Main Section*	Health-Anxiety	0–42	≥ 20 [47, 48] Health Anxiety	Not available	Worried thoughts and feelings about health states	14	4-point Likert- scale; Higher scores, more frequent symptoms	Past week
Patient Health Questionnaire- 9 (PHQ-9)	Depressive Disorder	0–27	≥ 10 [49] Major Depression	88, 85 [49]	Frequency of depressive symptoms	9	4-point Likert- scale; Higher scores, more frequent symptoms	Past 2 weeks
Generalised Anxiety Disorder 7-item Scale (GAD-7)	Generalised Anxiety Disorder	0–21	≥ 10 [50] Generalised Anxiety Disorder	89, 82 [50]	Frequency of anxious thoughts and feelings	7	4-point Likert- scale; Higher scores, more frequent symptoms	Past 2weeks
Obsessive-Compulsive Inventory Revised (OCI-R)	Obsessive Compulsive Symptoms	0–72	≥ 21 [51] Obsessive-Compulsive Disorder	65.6, 63.4 [51]	Subscales of obsessive and compulsive symptoms	18	5-point Likert- scale; Higher scores, greater agreement	Past month
Standardised Assessment of Personality – Abbreviated Scale (SAPAS)	Personality Disorder	0–8	≥ 4 [52] Personality Disorder	94, 85 [52]	Agreement with personality traits	8	Yes / No	In general
Alcohol Use Disorders Identification Test for Consumption (AUDIT-C)	Alcohol Misuse	0–12	≥4 in men and ≥ 3 in women [53] Hazardous alcohol use	Men: 86, 89 Women: 73, 91 [53]	Agreement with patterns of hazardous alcohol-use	5	Varies with each item within the scale – 5-severity categories	Varies with each item within the scale
Single-Question Screening Test for Drug Use	Drug Misuse	0–1	≥1 [54] Drug use disorder	100, 73.5 [54]	Frequency of illicit drug use or misuse of prescription medication.	1	Binary; None / once or more than once	Past year
Work and Social Adjustment Scale (WSAS)	Social and occupational function	0–40	≥21 Moderately severe psycho-pathology [33, 40]	–	Severity of impairment in work, home management, social activities, private leisure activities, close relationships.	5	9-point Likert scale; higher scores more impairment	In general
EuroQuol 5 Domains (EQ-5D-3 L UK adjusted index values)	Quality of life	−0.594 - 1	N/A	–	Severity of health-related quality of life in mobility, self-care, usual activities, pain, anxiety/depression.	5	3-point Likert scale; higher scores, greater impact on quality of life	In general

We also collected information on participants’ use of health care services over the preceding 3 months with questions modified from the Adult Service Use Schedule [55]. These included the numbers of participant reported inpatient hospital admissions, outpatient clinic and general practice appointments, attendances to the emergency department, and talking therapy sessions.

### Medical comorbidities

Respondents self-reported their medical co-morbidities as free-text responses. These were coded using the MedDRA international system by a medically trained clinician [56]. Comorbidities associated with an increased risk of hospitalisation or mortality from COVID-19 are reported by the QCOVID project, and are listed in Additional file 1: Appendix 2 [57]. This system was used to inform the British National Health Service on who should take particular care to isolate themselves, also known as ‘shielding’ [58].

### Data analysis

Data were managed and analysed with Stata/IC v16.1 (StataCorp LLC, College Station, TX). Respondents were required to complete each item within an assessment measure to be able to progress through the survey, hence there were no missing data points within variables. Descriptive statistics and the distribution of outcome measures are reported, and psychopathology assessment tool results were described alongside corresponding prevalence figures in the UK general population. Means were used as the measure of central tendency as variables all approximated a normal distribution taking kurtosis of a normal distribution as less than 3 and skew between − 1 and 1. Outliers were identified as outside three standard deviations from the mean. Comparisons between demographic variables and WSAS and ED-5Q-3 L index measures are made with t-tests, and Mann-Whitney-U tests were employed for single Likert-type protective behaviour items. All statistical tests were two-sided. In analysis, ‘washing behaviours’ were combined by mean averages of three Likert-type items: washing hands, clothes, and goods brought into the home. A series of Pearson’s correlation coefficients are reported between covariates and outcomes. Starting by including all independent variables we used backwards stepwise elimination multiple linear regression models with removal by significance testing at the alpha level of 0.05, to explore factors most associated with health behaviours, functional impairment and poor quality of life among those living with severe COVID anxiety. Continuous independent and dependent covariates were standardised by their z-scores to generate standardised regression coefficients in order to facilitate comparison. The continuous covariates included in the models were age, and the psychopathological screening tools: sHAI, PHQ-9, GAD-7, OCI-R, SAPAS and AUDIT-C. Binary coefficients were not standardised, and therein coefficients cannot be directly compared to those of continuous covariates. Binary factors included in the full models were: sex, having one or more at-risk health condition, previous COVID-19 infection, living alone, being in employment, from an at-risk ethnic group, living with someone thought to be vulnerable to COVID-19, and having had a loved one hospitalised by COVID-19. Assumptions of the linearity of residuals were confirmed graphically, and heteroskedasticity in all models was addressed by employing robust standard errors. The multicollinearity of all models were assessed and deemed acceptable by examining the variance inflation factors of independent variables, which were in each case approximately 1.

## Results

A total of 1068 respondents completed the initial screening questionnaire. After excluding 30 people who reported a history of psychosis, 4 who were not residents of the United Kingdom, 27 who did not complete all CAS items, and 14 who did not consent to participate (3 individuals with history of psychosis also did not consent to participate), 996 people provided full CAS scores. The mean CAS score was 7.6 (standard deviation = 5.68, skew = 0.67, kurtosis = 2.62), and Cronbach’s coefficient alpha of 996 participants was 0.91. In total 622 (62.4%) of those completing the CAS scored below 9; 306 (30.4%) scored 9 or more, met other eligibility criteria, and responded to the baseline assessment.

Of the 306 baseline study participants 246 (81.2%) were female, with a median average age of 41 (IQR 28–53; range 18–83). Age was positively skewed: while 20% of the sample was aged between 18 and 25, 13.9% were over 60. White British or Irish individuals made up 70.2% of the cohort, with 10.2% reporting being of White Other heritage, 7.6% South Asian, 4% Black, 4% as having a mixed ethnic heritage, 2.1% as Other ethnic group, and 2.3% did not report their ethnicity. Of those reporting their ethnicity, 52 were of a group with greater COVID-19 associated hospitalisation and mortality rates. A total of 139 (46.0%) people reported being employed, 15.6% unemployed, 13% were students, 3.0% were “furloughed” (receiving a proportion of their salary from the UK government, but not actively working), and the remainder were retired, homemakers or full-time carers.

Fifty-one of the 306 participants (16.9%) reported having previously had COVID-19, of which 34 were confirmed by PCR test or serum antibodies, and 6 had been hospitalised by the virus. Four in ten (39.4%) participants stated that they lived with someone thought to be vulnerable to COVID-19, and one in three (32.3%) reported having a close family member or friend who was admitted to hospital as a result of COVID-19.

### Physical and mental health comorbidity

In total 192 (60.3%) of the sample reported at least one comorbidity, including 117 (40.3%) who were multimorbid living with 2 or more reported health conditions, and 18 (6.2%) participants who were living with five or more. The most common reported comorbidities were anxiety (62; 21.2%), asthma (40; 13.7%), depression (36; 12.3%), hypertension (26; 8.9%), fibromyalgia (18; 6.2%), diabetes mellitus (18; 6.2%), irritable bowel syndrome (13; 4.5%) and non-inflammatory arthritis (13; 4.5%). One quarter (76; 26.0%) of the cohort reported a comorbidity associated with an increased risk of hospitalisation and mortality from COVID-19. These most commonly were asthma (40; 13.7%), diabetes mellitus (18; 6.2%), rheumatoid arthritis (5; 1.7%), chronic-obstructive pulmonary disease (4; 1.4%), active cancer (3; 1.0%), HIV infection (3; 1.0%), cardiac arrythmia (3; 1.0%), and obesity (3; 1.0%). Existing diagnoses of non-psychotic mental health disorders were disclosed by 89 participants (30.6%).

Summary statistics of covariates and outcomes along with scales’ coefficient alpha and comparisons to UK population averages are presented in Table 2. Over 90% of the sample met scores diagnostic of generalised anxiety disorder, over 80% for depression, around 70% met threshold scores for obsessive-compulsive disorder and health anxiety, and 60% for personality disorder. Almost four in ten reported hazardous alcohol use, and 8.8% of the group disclosed use of an illicit drug or unprescribed use of prescription-only medication on at least one occasion in the preceding year. Eleven participants (3.6%) did not score above threshold on any of the co-morbid psychopathology scales.

**Table 2:** Summary statistics for outcomes and covariates

	Variable	N =	Mean (s.d)	Min - Max	Coef. alpha	Skew	Kurtosis	% of sample above cut-off	% estimated prevalence in UK population / UK average
Inclusion criteria	CAS	306	12.4 (3.00)	9–20	–	0.92	3.1	100	Unavailable
Covariates	sHAI	290	23.5 (7.0)	6–40	0.87	−0.1	2.7	69.3	19.8^a^ [59]
	PHQ-9	289	15.7 (5.4)	0–26	0.84	−0.14	2.4	85.5	3.3 [60]
	GAD-7	295	15.5 (3.96)	4–21	0.82	−0.58	2.6	91.5	5.9 [60]
	OCI-R	285	30.1 (15.3)	0–72	0.92	0.31	2.4	68.1	1.3 [60]
	SAPAS	284	4.2 (1.84)	0–8	0.55	−0.03	2.4	63	13.7 [60]
	AUDIT-C	285	2.7 (2.94)	0–12	0.80	0.9	3.0	Men 35.7	16.6 [60]
								Women 37.0	
Outcomes	WSAS	288	21.2 (7.52)	0–39	0.71	−1.1	2.6	52.4	Unavailable
	EQ-5D-3 L index scores	287	0.498	−0.536 - 1	–	−0.57	2.4	–	0.856 [61]
Health behaviours	Hand washing	289	3.0 (0.68)	0–4	–	−0.39	3.3	–	–
	Washing clothes	289	2.33 (1.06)	0–4	–	0.17	1.8	–	–
	Disinfecting deliveries	289	2.6 (1.12)	0–4	–	−0.04	1.6	–	–
	Leaving home	289	2.76 (0.71)	0–4	–	−0.43	3.2	–	–
	Consuming media	289	3.36 (0.98)	0–5	–	−0.17	2.5	–	–

^a^The prevalence of health anxiety in UK general medical clinics - estimates for the UK general population are unavailable. s.d. = standard deviation, coef. alpha = Cronbach’s coefficient alpha

Despite some evidence for the direct effects of COVID-19 with the onset of new psychopathology [10], using two-tailed t-tests there was no evidence to suggest that having previously had COVID-19 versus never having been infected, or being hospitalised versus having COVID-19 but not hospitalised, were associated with differences in scores on any of the psychopathology measures applied to this sample.

Ten participants (4.4%) reported having had a period of inpatient hospital care within the last 3 months, and 73 (30.8%) reported at least one outpatient clinic appointment (median number of appointments = 1). Over the same time period 29 people (13.0%) had at least one attendance to an emergency department (median number of attendances = 1), and 196 (72.9%) had an appointment with their General Practitioner (median GP visits = 2). Finally, 95 respondents (39.1%) reported at least one session of a talking therapy within the past 3 months (median number of sessions = 4).

### COVID health behaviours

One in five participants (22.2%) reported being in a constant state of worry about the pandemic, and over 90% of this sample of people living with severe COVID anxiety confirmed worrying about COVID at least daily. For 11.8%, their consumption of news reports about COVID-19 was ‘constant’, and a further 34.6% consumed related media multiple times per day.

Regarding the three measured avoidant behaviours, a majority of respondents (69.55%) did not leave their homes on a daily basis, with 11.0% percent of the sample reporting never leaving their homes. Thirty-eight percent of respondents reported that they bought all their food online due to concerns about the pandemic, compared with 13.9% who shop exclusively online for the convenience. For an additional 52 participants (18.0%) other people bought all food for them. Of the 97 respondents with children, 57 (58.8%) reported that they went to school as normal, 21 (21.6%) reported that their children did not go to school because of other factors such as the school being closed or home-schooling, and 19 (19.6%) respondents disclosed not sending their children to school, despite it being open, due to their concerns about COVID-19.

Preventive behaviours were also common with 31.5% of participants reporting washing, disinfecting or discarding all goods brought into the home - 19.0% did not do so at all. Almost all (98.0%) individuals had increased the frequency of their hand washing, with one in five (20.8%) washing their hands ‘constantly’. Seventy-two percent of the sample washed their clothes more frequently than before the pandemic, including 17.3% who washed every piece of clothing each time it had been worn outside.

Bivariate analyses of factors associated with health behaviours are presented in Tables 3 and 4 reports the correlation coefficients between health behaviours and psychopathological assessment scores. Table 5 reports parsimonious predictive multiple linear regression modelling of the contribution of demographic and psychological domains in social and occupational functioning, quality of life and COVID health behaviours.

**Table 3:** Group mean comparisons of protective behaviours, functional impairment and quality of life measures

	Protective behaviours							Functional impairment			Quality of life		
	n=	Consuming news media	p=	Staying home	p=	Washing behaviours	p=	n=	WSAS	p=	n=	EQ5D-3 L	p=
Female	234	3.34	0.57	2.79	0.11	2.69	0.09	234	20.9	0.16	234	0.487	0.22
Male	55	3.44		2.64		2.5		54	22.5		54	0.548	
At-risk ethnicity	50	3.18	0.19	2.60	0.20	2.83	0.10	49	23.5	0.02	49	0.583	0.05
Not at-risk	234	3.40		2.78		2.61		234	20.8		234	0.481	
Employed	129	3.45	0.16	2.58	< 0.001	2.57	0.14	129	21.4	0.54	128	0.561	0.005
Unemployed	159	3.29		2.89		2.72		158	20.		158	0.449	
Lives alone	46	3.4	0.73	2.89	0.09	2.55	0.46	45	23.7	0.013	45	0.362	0.003
Lives with others	242	3.35		2.72		2.67		242	20.7		241	0.525	
Has had COVID-19	46	3.04	0.02	2.7	0.77	2.53	0.24	46	21.1	0.96	46	0.471	0.53
No previous COVID-19	242	3.43		2.76		2.67		241	21.2		240	0.504	
≥1 at-risk health condition	73	3.3	0.78	2.95	0.002	2.87	0.003	73	20.3	0.25	72	0.404	0.005
No at-risk health conditions	214	3.38		2.68		2.58		213	21.6		213	0.531	
Lives with someone vulnerable	114	3.59	0.003	2.83	0.26	2.53	0.001	114	22	0.11	114	0.477	0.36
Does not live with some vulnerable	174	3.21		2.7		2.84		173	20.6		172	0.514	
Loved one hospitalised by COVID	95	3.34	0.84	2.75	0.88	2.68	0.81	95	22.4	0.042	95	0.523	0.39
No loved one hospitalised by COVID	193	3.37		2.75		2.64		192	20.5		191	0.487

**Table 4:** Correlations coefficients between psychopathology scales and outcomes

Mental health	Consuming COVID media	Staying home	Washing behaviours	Work and Social Adjustment Scale (WSAS)	EuroQol 5 Dimensions – index scores (EQ5D-3 L)
Covid Anxiety Scale (CAS)	0.07	0.06	0.16*	0.17**	−0.17**
Short Health Anxiety Inventory (sHAI)	0.18**	0.15*	0.39***	0.24***	−0.40***
Patient Health Questionnaire – 9 (PHQ-9)	0.14*	0.17**	0.03	0.48***	−0.48***
Generalised Anxiety Disorder Assessment – 7 (GAD-7)	0.19***	0.11	0.17**	0.29***	−0.33***
Obsessive-Compulsive Inventory – Revised (OCI-R)	0.03	0.14*	0.29***	0.32***	−0.19**
Standardised Assessment of Personality – Abbreviated Scale (SAPAS)	0.05	0.16**	0.02	0.11	−0.40***
Alcohol Use Disorder Identification Test of Consumption (AUDITC)	0.07	−0.04	−0.19**	− 0.03	0.007

*p < 0.05; **p < 0.01; ***p < 0.001

**Table 5:** Parsimonious multiple linear regression models with standardised coefficients predicting functional impairment, quality of life, and protective behaviours

**Consuming news reports**	β	SE	p	CI
Vulnerable cohabitant^a^	0.35	0.16	0.003	0.12–0.58
GAD-7	0.15	0.06	0.008	0.04–0.26
Previous COVID-19^a^	−0.37	0.17	0.029	−0.71 - -0.03
**Staying home**	β	SE	p	CI
≥1 at-risk health condition^a^	0.31	0.12	0.011	0.07–0.55
PHQ-9	0.19	0.06	0.002	0.07–0.31
In employment^a^	−0.41	0.12	< 0.001	−0.63 - -0.19
**Washing behaviours**	β	SE	p	CI
OCI-R	0.33	0.06	< 0.001	0.22–0.47
sHAI	0.32	0.05	< 0.001	0.23–0.44
≥1 at-risk health condition^a^	0.30	0.12	0.01	0.16–0.65
Vulnerable Cohabitant^a^	0.27	0.11	0.01	0.07–0.48
AUDIT-C	−0.11	0.05	0.04	−0.21 - -0.001
SAPAS	−0.12	0.06	0.04	−0.24 - -0.04
PHQ-9	−0.17	0.06	0.005	−0.3 - -0.05
Male^a^	−0.33	0.13	0.01	0.11–0.63
**WSAS**	β	SE	p	CI
PHQ-9	0.45	0.06	< 0.001	0.34–0.56
Of an at-risk ethnic group^a^	0.33	0.14	0.022	0.05–0.62
Loved one hospitalised^a^	0.23	0.1	0.04	0.01–0.44
OCI-R	0.13	0.06	0.03	0.01–0.25
**EQ-5D-3 L**	β	SE	p	CI
Living alone^a^	−0.36	0.16	0.022	−0.67 - -0.05
PHQ-9	−0.34	0.05	< 0.001	−0.45 - -0.23
sHAI	−0.20	0.05	< 0.001	−0.29 - -0.1
≥1 at-risk health condition^a^	−0.23	0.11	0.04	−0.45 - -0.01
SAPAS	−0.15	0.05	0.002	−0.25 - -0.06
Of an at-risk ethnic group^a^	0.32	0.15	0.026	0.038–0.61
In employment^a^	0.32	0.09	0.001	0.13–0.5

F(3, 283) = 7.4, adjusted model *R*^2^ = 0.093, *p* < 0.001

F(3, 282) = 8.68, adjusted model *R*^2^ = 0.10, *p* < 0.001

F(8, 273) = 15.25, adjusted model *R*^2^ = 0.30, *p* < 0.001

F(4, 270) = 28.5, adjusted model *R*^2^ = 0.29, *p* < 0.001

F(7, 267) = 30.37, adjusted model *R*^2^ = 0.38, *p* < 0.001

^a^binary factor. *GAD-7 *General Anxiety Disorder Assessment 7, *PHQ-9 *Patient Health Questionnaire 9,* OCI-R *Obessive-Compulsive Inventory Revised, *sHAI *Health Anxiety Inventory Short Form, *AUDIT-C * Alcohol Use Disorders Identification Test Consumption, *SAPAS *Standardised Assessment of Personality Abbreviated Scale

Living with someone thought to be vulnerable to COVID-19 (*p* < 0.001), and increasing generalised (*r =* 0.19, *p* < 0.001) and health anxiety symptoms (*r =* 0.18, *p* < 0.001) both predicted reports of increased consumption of COVID-19 related media, however health anxiety lost its significance in multivariate modelling. Those who reported previous infection with SARS-CoV2 consumed significantly fewer media reports on the subject.

People with severe COVID anxiety were more likely to stay at home if they have an at-risk health condition (*p* < 0.001), greater depressive symptoms (*r* = 0.17, *p* < 0.01) and disordered personality traits (*r* = 0.16, *p* < 0.01). Whereas those in employment were significantly more likely to leave their home (*p* < 0.001). In multiple regression modelling, disordered personality traits were no longer significant. Changes by each standard deviation on the PHQ-9 were significant in predicting a change by 19% of one standard deviation on the staying at home Likert scale, when controlling for other factors (β = 0.19; CI 95% 0.07–0.31).

Increasing obsessive-compulsive (β = 0.33; CI 95% 0.22–0.47), and health anxiety symptoms (β = 0.31; CI 95% 0.23–0.44), both strongly predicted washing behaviours in this severely COVID anxious sample, as did living with someone thought to be vulnerable to COVID-19 (β = 0.27; CI 95% 0.07–0.48), and having an at-risk health condition (β = 0.3; CI 95% 0.16–0.65). Increasing depressive symptoms, personality disorder traits, alcohol use and being male on the contrary predicted significantly fewer washing behaviours in this sample.

There were no meaningful associations between participant age or gender and any health behaviours.

### Social and occupational dysfunction

Of 288 participants providing WSAS scores, 15 (5.6%) had mild social or occupational dysfunction, 122 (42.7%) moderate dysfunction and 151 (52.3%) severe dysfunction (including 36 (12.5%) of the sample scoring over 30). Individuals with severe COVID anxiety from an ethic group at increased risk from COVID-19 were significantly more likely to report severe social impairment (*p* = 0.001), as were people who lived alone (*p* = 0.013) or had a close family member or friend hospitalised by COVID-19 (*p* < 0.05) (Table 3). Depressive symptom scores were most strongly correlated with functional impairment (*r* = 0.48, *p* < 0.001) (Table 4). In multiple regression modelling co-morbid depressive (β = 0.45; 95% CI 0.34–0.56; *p* < 0.001) and OCD symptoms (β = 0.13; 95% CI 0.01–0.25; *p* < 0.001) were each predictive of a significant increase in functional impairment scores on the WSAS. Being of an at-risk ethnic group and having a loved one hospitalised by COVID were also significant predictors of worse social and occupational functioning in multivariate modelling.

### Quality of life

Only 12 respondents (4.18%) described living in the best imaginable health state. Problems were reported in mobility by 28.5% of respondents, self-caring activities by 22.6%, usual activities by 52.8%, pain or discomfort by 57.3%, and being anxious or depressed by 93.4%. The most common EQ-5D-3 L profiles were: 11112 (15.7%), 11,122 (10.1%), 11,113 (6.6%), and 11,222 (5.6%). Thirty (10.4%) people gave quality of life scores less than 0, conceptualised as living in a health state worse than death, with the lowest score, − 0.536 representing 33,233. Depressive symptomatology was most strongly negatively correlated with EQ-5D-3 L index scores, (*r* = − 0.48, *p* < 0.001), followed by increasing health anxiety and disordered personality traits (Table 4). Of the factors explored in multiple regression modelling, living alone, co-morbid depressive, health anxious and personality disorder symptoms, and having an at-risk health condition were identified as significant predictors of a worse quality of life (Table 5). Being in employment and of an at-risk ethnic group were both significant predictors of a better quality of life for people with severe COVID anxiety.

## Discussion

This article characterises a sample of over 300 individuals recruited from across the United Kingdom living with severe COVID anxiety. These results highlight first, the notable functional impairment, poor quality of life and the use of protective behaviours among people with severe COVID anxiety, secondly, high rates of psychiatric co-morbidity, and third, demographic and clinical factors associated with worse outcomes. These findings are unique, because while a number of demographic factors associated with the employment of protective behaviours and anxiety have been suggested from work in previous pandemics [2], there have been no studies from previous pandemics examining the links between individuals with severe pandemic-related anxiety and their demographic characteristics, protective behaviours, quality of life or daily functioning.

More than half the sample reported severe social dysfunction, comparable to people referred to secondary care mental health services [62–64] and those starting psychological treatments in the UK [65]. Among people with severe COVID anxiety, living alone predicted a worse quality of life after adjusting for other demographic and psychopathological factors, correspondingly a better quality of life was observed among those in employment. Interestingly, individuals from an ethnic background at an increased risk from COVID-19 reported a significantly poorer functional status, but better quality of life. As with other studies on the topic, a mediating role of social support may play a part in explaining these observations [15, 66, 67].

Health behaviours enacted to an excessive degree were common in the whole sample. Hand washing and consuming COVID-19 news reports were described as “constant” by over 20 and 10% respectively. Washing/disinfecting all food, letters or parcels coming into homes was reported by 30, and 45% wash their clothes a lot more since the start of the pandemic, both of which UK national public health guidance suggest are unnecessary to prevent the spread of COVID [68, 69]. A large majority of the UK general population (93%) has consistently reported engaging in at least one government recommended health behaviour, with the largest uptake being 83% reporting increasing the frequency of their hand washing [70], in comparison to 98.0% reporting doing so in this severely anxious sample. Likewise, during the data collection period a rolling average of 5% of the UK population reported not having left their home in the past 7 days [71] a figure which is doubled in this sample. The observation that 20% of families with children in this sample do not send them to school because of fears of the coronavirus adds to evidence of the impact of parental COVID anxiety on their children [72, 73].

People with severe COVID anxiety who took part in the study had poor mental health, with high levels of anxiety, depressive and obsessive-compulsive symptoms, and disordered personality traits. While alcohol use has increased among UK adults during the pandemic, respondents to this survey report a rate of hazardous alcohol use double the pandemic national average for both men and women [74, 75]. The prevalence of self-reported physical health conditions that placed people at higher risk from COVID was similar in the sample to that of UK general population estimates at around a quarter, while the proportion of people stating that they had asthma was twice that seen in the general population [76, 77]. Although little UK data is available, it also appears, compared to figures from the United States, that the prevalence of self-reported fibromyalgia or chronic fatigue syndrome were notably higher in this sample than expected [78]. With 40% living with multimorbidity, 30% with mobility problems, 60% living with pain, and over 20% limited in their self-care, this sample reveals a highly morbid population, with considerable healthcare use.

Living with an at-risk health conditions appears to increase the likelihood of developing COVID anxiety [79]. The present study however did not demonstrate a difference in the severity of severe COVID anxiety among those with and without an at-risk condition (*p* = 0.95). Yet these results do show that the vulnerable group of people with both severe COVID anxiety and an at-risk health condition experience a poorer quality of life and employ more protective behaviours than those without an at-risk health condition, highlighting the complex interplay between physical health and psychological phenomena in COVID anxiety: this group may therefore require extra consideration in future research.

This sample also identified 51 individuals who live with severe COVID anxiety reporting having already been infected by the virus. Having had COVID was not associated with differences in functional impairment or quality of life, albeit these individuals did report less frequently consuming COVID-19 related news items. The concept of health anxiety in fear of disease recurrence is not uncommon and demonstrated in patients of varying chronic disease states [80]. These present data appear to suggest this process also occurs with COVID anxiety for some individuals.

Finally, the finding that independently depressive symptoms best explained functional impairment and poor quality of life among the psychopathological factors studied in people living with severe COVID anxiety is an important one. With a shortage of evidence-based approaches for addressing severe COVID anxiety and improving daily life for those affected, interventions might herein look towards targeting depressive psychopathology. Likewise, these data add evidence to suggest that particular attention to people with severe COVID anxiety who live alone and with at-risk health conditions may improve their social functioning and quality of life. There are many potential explanations of these observations including that psychopathological and social aspects might interact with each other in more complex ways, as well as with social dynamics of the pandemic.

### Strengths and limitations

As far as we are aware this is the first time that a sample of UK adults with severe COVID anxiety have been characterised. Despite using a broad range of methods to recruit the study sample, as this was not a random sample we cannot be sure that these results are generalisable to everyone with severe COVID anxiety.

The cross-sectional design means that we are not able to explore the causal network of these observations. For instance, previous longitudinal data has pointed to pre-existing poor mental health as a risk factor for COVID anxiety [28–30]. So while we have demonstrated high rates of depression, generalised anxiety, health anxiety, and OCD among people with severe COVID anxiety these data cannot confirm whether those conditions pre-existed and predisposed people to COVID anxiety, or whether instead COVID anxiety and resulting protective behaviours contributed to the development of co-morbid psychopathology. Moreover, the Coronavirus Anxiety Scale, which prioritises physiological responses to the virus cannot differentiate between underlying somato-psychological processes - autonomic responses, panic, post-traumatic re-experiencing, somatic symptoms of anxiety, among others – and herein severe COVID anxiety as measured by the CAS is likely to represent individuals with a multitude of psychopathological processes.

All of our data were collected between January and September 2021, a period which started in the middle of the second wave in the UK and ended during the third wave and delta variant becoming prominent. Through this time there were 2 months of national lockdown, in which people were able to form a ‘social bubble’ mutually with one other household and could leave their home for essential travel only. Restrictions otherwise varied greatly over the data collection period and by location within the UK. We did not collect data on participants’ primary region or city of residence and therefore cannot model for the effects of geographical variation in lockdown restrictions over the collection period. Several studies have shown that periods of lockdown and other time-dependent social restrictions affect levels of anxiety and functional impairment which may be a strong unaccounted mediating factor in our results [81].

Finally, all the data we collected were self-reported. Where possible we used measures which are reliable and have been widely used in previous community surveys. However, the psychometric properties of other measures we used, such as those for examining COVID-related behaviours, have not been tested. Self-report items also meant that we were also unable to objectively assess the clinical severity of several conditions, particularly asthma, which would have only qualified as an at-risk condition if severe. Some participants in the at-risk group are therefore likely to not be at an objectively increased risk, although we suspect many individuals, having reported their medical conditions among other questions concerning risk factors for COVID, did so with the impression that their condition did increase their risk. In this sense reporting of an at-risk group becomes a proxy measure of perception of risk from COVID-19 rather than necessarily objective risk.

## Conclusions

This sample of UK adults living with severe COVID anxiety experience significant impacts to their daily functioning equivalent to that of patients under secondary mental health services, and a quality of life notably worse than the general population. Health behaviours are near universal in the sample, and are for a sizeable minority enacted in excess of national guidance. This study also contributes to evidence that children of severely COVID anxious parents are also adversely affected. Depressive symptoms appear to drive much of the associated poor quality of life and functional impairment, and interventions which target these symptoms may therefore lead to better outcomes for these individuals. People with severe COVID anxiety and living with at-risk medical conditions also appear to experience a disproportionately poorer quality of life and are more likely to employ protective behaviours to an unhelpful degree: particular attention ought be placed on this group. Meanwhile further research is needed to establish the course of severe COVID anxiety, factors associated with its improvement or maintenance, and the impact of interventions aimed at helping people who experience this distress.

## Supplementary Information

**Additional file 1.**

## Data Availability

Data relating to the present analysis are available from the corresponding author on reasonable request. In due course the full COVID Anxiety Project dataset will be made publicly assessable.

## Abbreviations

CAP: COVID-19 Anxiety Project
CAS: COVID Anxiety Scale
EQ-5D-3 L: EuroQuol 5 Domains – 3 item Likert scale
GAD-7: Generalised Anxiety Disorder – 7 items
HAI: Health Anxiety Inventory
NHS: National Health Service
PHQ-9: Patient Health Questionnaire – 9 items
QoL: Quality of Life
OCD: Obsessive-Compulsive Disorder
OCI-R: Obsessive-Compulsive Inventory – Revised
SAPAS: Structured Assessment of Personality Abbreviated Scale
UK: United Kingdom
WSAS: Work and Social Adjustment Scale
