# Supplementary material for: The association of severe COVID anxiety with poor social functioning, quality of life, and protective behaviours among adults in United Kingdom: a cross-sectional study
Source: BMC Psychiatry. 2023 Feb 21;23:117. doi: 10.1186/s12888-023-04595-1 (PMC9943584; doi:10.1186/s12888-023-04595-1)
Supplement: Supplementary file 1 — Additional file 1. [file 12888_2023_4595_MOESM1_ESM.docx]

**Appendix 1: COVID-19 protective behaviour items.**

| **Question** | **Options** |
| --- | --- |
| How much of your time have you spent worrying about COVID-19? (Ordinal) | None of the time;  Some of the time (less than daily);  Every day;  Several times a day;  Constantly |
| How much time have you spent reading/watching news (TV/online/social media) about COVID-19? (Ordinal) | None of the time;  Some of the time (less than daily);  Every day;  Several times a day;  Constantly |
| How many times did you leave your home? (Ordinal) | More than once a day;  Every day;  Some of the time (less than daily);  None of the time; |
| Do you have children living with you? If so; (Categorical) | Yes, children are able to attend and have been attending school  Yes, children are able to attend school but have not attended due to concerns regarding COVID-19  Yes, but the children are not able to attend school (due to provision or other factors)  No children |
| Regarding food shopping; (Categorical) | You buy all your own food in stores;  You buy all your food online for convenience;  Other people buy all your food for you;  You buy all your food online due to concerns going to stores |
| When food, letters or parcels come into your house, was there ever an occasion when you washed or discarded items because of possible contamination with COVID-19 virus? (Ordinal) | None of the time;  Yes, one or more items;  Yes, most items  Yes, all items |
| On average, how often do you wash your hands? (Ordinal) | Same as prior to the start of COVID-19;  Slightly more often than prior to the start of COVID-19;  A lot more often than prior to the start of COVID-19;  Constantly; |
| On average, how often are you washing your clothes? (Ordinal) | Same as prior to the start of COVID-19  Slightly more often than prior to the start of COVID-19  A lot more often than prior to the start of COVID-19  Each time an item is worn outside |

**Appendix 2. At risk disease states, taken from QCOVID risk** **tool** [58].

Obesity, diabetes mellitus, chronic kidney disease, sickle cell disease, severe combined immunodeficiency syndrome, human immunodeficiency virus (HIV) or other acquired severe immunodeficiency, learning disability, current cancer of the blood or bone marrow, solid organ transplant, oral or lung cancer, asthma, cystic fibrosis, bronchiectasis, alveolitis, pulmonary hypertension, pulmonary fibrosis, chronic obstructive pulmonary disease (COPD), Parkinson’s disease, epilepsy, dementia, motor neurone disease, multiple sclerosis, myasthenia, Huntington’s disease, cerebral palsy, congenital heart problem, coronary heart disease, stroke or transient ischaemic attack (TIA), atrial fibrillation, heart failure, peripheral vascular disease, thrombosis or pulmonary embolus, severe mental illness, cirrhosis of the liver, rheumatoid arthritis, systemic lupus erythematosus, osteoporotic fracture of the hip, wrist, spine or humerus
